# Limited accessibility to designs and results of Japanese large-scale clinical trials for cardiovascular diseases

**DOI:** 10.1186/1745-6215-12-96

**Published:** 2011-04-14

**Authors:** Hiroshi Sawata, Kenji Ueshima, Kiichiro Tsutani

**Affiliations:** 1 Department of Drug Policy and Management, Graduate School of Pharmaceutical Sciences, The University of Tokyo, 7-3-1 Hongo, Bunkyo-ku, Tokyo, Japan; 2 EBM Research Center, Kyoto University Graduate School of Medicine, Yoshida Konoe-cho, Sakyo-ku, Kyoto, Japan

## Abstract

**Background:**

Clinical evidence is important for improving the treatment of patients by health care providers. In the study of cardiovascular diseases, large-scale clinical trials involving thousands of participants are required to evaluate the risks of cardiac events and/or death. The problems encountered in conducting the Japanese Acute Myocardial Infarction Prospective (JAMP) study highlighted the difficulties involved in obtaining the financial and infrastructural resources necessary for conducting large-scale clinical trials. The objectives of the current study were: 1) to clarify the current funding and infrastructural environment surrounding large-scale clinical trials in cardiovascular and metabolic diseases in Japan, and 2) to find ways to improve the environment surrounding clinical trials in Japan more generally.

**Methods:**

We examined clinical trials examining cardiovascular diseases that evaluated true endpoints and involved 300 or more participants using Pub-Med, Ichushi (by the Japan Medical Abstracts Society, a non-profit organization), websites of related medical societies, the University Hospital Medical Information Network (UMIN) Clinical Trials Registry, and clinicaltrials.gov at three points in time: 30 November, 2004, 25 February, 2007 and 25 July, 2009.

**Results:**

We found a total of 152 trials that met our criteria for 'large-scale clinical trials' examining cardiovascular diseases in Japan. Of these, 72.4% were randomized controlled trials (RCTs). Of 152 trials, 9.2% of the trials examined more than 10,000 participants, and 42.8% examined between 1,000 and 10,000 participants. The number of large-scale clinical trials markedly increased from 2001 to 2004, but suddenly decreased in 2007, then began to increase again. Ischemic heart disease (39.5%) was the most common target disease. Most of the larger-scale trials were funded by private organizations such as pharmaceutical companies. The designs and results of 13 trials were not disclosed.

**Conclusions:**

To improve the quality of clinical trials, all sponsors should register trials and disclose the funding sources before the enrolment of participants, and publish their results after the completion of each study.

## Background

Large numbers of clinical and non-clinical investigations are required to obtain evidence to improve the treatment of patients. This evidence can benefit the medical practice of health care providers by informing treatment guidelines and providing the rationale on which to make treatment decisions.

Clinical trials are necessary for producing appropriate clinical evidence. In clinical trials examining cardiovascular diseases, large-scale clinical trials with thousands of participants are often required to evaluate the risks of cardiac events and/or death, because it is necessary to evaluate the incidence of cardiovascular events that are relatively uncommon. Such clinical trials provide evidence about the most appropriate treatment regimen for preventing cardiovascular and metabolic diseases.

In the 1970s, researchers began to conduct clinical trials in Western countries, with the incidence of cardiovascular events as an endpoint [1,2]. Although the large-scale clinical trials in Japan were occurred approximately 10 years ago, the number of large-scale clinical trials has increased. Recently, a large number of clinical trials evaluating the incidence of cardiac events and/or death using hard endpoints have been conducted in the field of cardiovascular and metabolic medicine.

The Japanese Acute Myocardial Infarction Prospective (JAMP) study was the first non-pharmaceutical company-supported multicenter trial of a medication in Japan, and the results have already been reported [3]. Briefly, this randomized parallel-group study was carried out across 48 institutions from 1993 to 2000. The primary endpoint of the study was a composite of cardiac events involving at least one of the following: cardiac or non-cardiac death, recurrent non-fatal myocardial infarction, coronary revascularization, and hospitalization because of worsening angina or congestive heart failure. In total, 888 of 1,163 participants with acute myocardial infarction (AMI) were eligible for the full analysis set (FAS). Patients were randomly assigned to two groups; 422 received angiotensin-converting enzyme (ACE) inhibitors and 466 did not receive ACE inhibitors. The mean follow-up period was 5.8 years. The JAMP study group concluded that no significant improvement in outcome was associated with ACE inhibitor administration in subjects who survived AMI in a Japanese study population.

Following the JAMP study, we conducted a review to highlight important issues regarding large-scale clinical trials. The major issues revealed by our review were the funding sources and infrastructure surrounding clinical trials. Financial and infrastructural resources must be maintained for clinical trials to be conducted appropriately. However, a high investigation cost is required for this, and obtaining adequate funding is critical for conducting clinical trials. The infrastructural environment necessary for clinical trials is currently inadequate, although the situation is improving, with efforts being made by government, medical institutions and other organizations. At present, Japanese clinical trials are typically funded by various sources, including public agencies, private companies and foundations. However, some researchers have suggested that industry-funded studies are likely to be affected by biases in their results and interpretations [4-10]. For example, Ridker, et al. reported that cardiovascular trials funded by for-profit organizations reported between 2000 and 2005, were more likely to report positive findings than those funded by not-for-profit organizations [4]. Given the current research situation in the medical and pharmaceutical industries, it is not possible to completely avoid conflicts of interests among researchers. Recently, the International Committee of Medical Journal Editors (ICMJE) requested that sponsors disclose certain information regarding trial management, including funding sources [11]. However, there are currently no comprehensive regulations for managing conflicts of interest.

The current study had two main objectives:

1) To clarify the current funding and infrastructural environment surrounding large-scale clinical trials in cardiovascular and metabolic diseases in Japan

2) To find more general ways to improve the environment surrounding clinical trials in Japan.

## Methods

Our search covered all large-scale clinical trials whose primary endpoints were true endpoints in clinical trials examining cardiovascular and metabolic diseases. A true endpoint was defined as an endpoint consisting of cardiovascular events, such as myocardial infarction, chronic heart failure, ischemic heart attack, and/or death. We defined 'large-scale clinical trials' as trials where the target number of participants or enrolled number of participants was 300 or more. If a trial was discontinued before enrolling 300 participants, but the planned number of participants was 300 or more, this trial was also regarded as 'large-scale clinical trial'. We searched for clinical trials using PubMed, Ichushi (Japana Centra Revuo Medicina by the Japan Medical Abstracts Society, a non-profit organization), URL: http://login.jamas.or.jp/), websites of related medical societies, University Hospital Medical Information Network (UMIN) Clinical Trials Registry (URL: http://www.umin.ac.jp/ctr/index-j.htm), and clinicaltrials.gov (URL: http://www.clinicaltrials.gov/).

We conducted searches at three different times. The first search was carried out in 2004, with a cut-off date of 30 November, 2004. The second and third searches were conducted in 2007 and 2009, with cut-off dates on 25 February, 2007 and on 25 July, 2009, respectively. The second search was conducted to evaluate the changes in the environment surrounding clinical trials in Japan after improving the awareness of conflicts of interest in 2005-2006. The third search was conducted to evaluate the impacts of some scandals regarding clinical researches that were reported by the media in Japan in 2007-2008.

For all clinical trials that met the criteria described above, we recorded the 1) sponsor, 2) objectives of trial, 3) design (randomized clinical trial or non-randomized clinical trial), 4) interventions, 5) chief investigator, 6) contact address, 7) starting year of the trial, 8) duration of the trial/ending year of the trial, 9) number of enrolled participants or target number of participants, 10) results of trial, 11) publications of results or methods of trial, 12) funding agencies, and 13) others. Here, we defined a 'sponsor' as 'an individual, company, institution or organization that took responsibility for the initiation, management and/or financing of a clinical trial' [12-14].

## Results

### 1) Screening of large-scale clinical trials in cardiovascular diseases

We found a total 152 trials conducted in Japan that met our criteria for 'large-scale clinical trials' examining cardiovascular diseases. Sixty-four trials were found in the search conducted on 30 November, 2004, 53 additional trials were found on 25 February, 2007, and 35 additional trials were found on 25 July, 2009.

### 2) Trial design (RCT/non-RCT) and number of participants

We categorized the trials as randomized controlled trials (RCTs) or non-randomized controlled trials (non-RCTs) according to their design, as shown in Table 1. 72.4% (110/152) of the trials were RCTs, and 27.6% (42/152) were non-RCTs. Examining the numbers of participants revealed that 9.2% (14/152) of the trials examined more than 10,000 participants, 42.8% (65/152) examined between 1,000 and 10,000 participants, and 42.8% (65/152) examined less than 1,000 participants. 28.6% (4/14) of the trials with 10,000 participants or more were RCTs, 70.8% (46/65) of the trials with 1,000 to 10,000 participants, and 81.5% (53/65) of the trials with less than 1,000 participants were RCTs. This result indicated that the proportion of RCTs tended to be higher in trials with a lower number of participants.

**Table 1:** Numbers of trials by number of participants and trial design (RCT or non-RCT)

Number of participants	Number of trials				Proportion of RCT (RCT/Total)
		
	RCT*	Non-RCT*	Total		
≥10,000	4	10	14	(9.2%)	0.286
1,000-10,000	46	19	65	(42.8%)	0.708
<1,000	53	12	65	(42.8%)	0.815
Unknown^†^	7	1	8	(5.3%)	-

Total	110	42	152	(100%)	0.724

* RCT: randomized controlled trial, Non-RCT: non-randomized controlled trial

† Eight multi-national trials where the number of Japanese participants was not known were categorized as 'unknown'.

### 3) Number of trials by starting year

**Figure 1:**
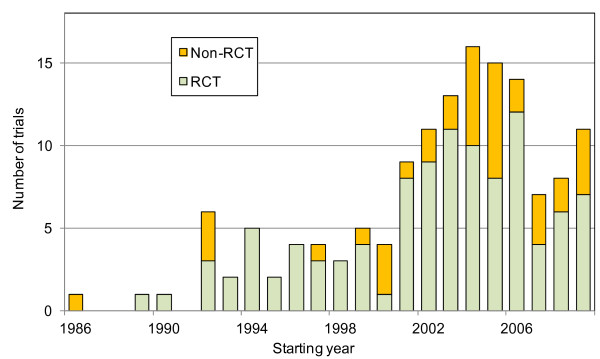
Number of trials by starting year and trial design (RCT or non-RCT). RCT: randomized controlled trial, Non-RCT: non-randomized controlled trial. Ten clinical trials (six RCTs and four non-RCTs) whose starting years were unknown were not counted.

We counted the number of trials according to the starting year and trial design (Figure 1). From 1992, several large-scale clinical trials were started each year. After 2001, the number of large-scale clinical trials markedly increased to more than 10 per year. In 2004, the number of large-scale clinical trials peaked, with 16 trials started within a year. In 2007, the number suddenly decreased to only seven, then increased again after 2007.

### 4) Number of trials by target disease

We categorized the numbers of trials by target disease (Table 2). Large-scale clinical trials examining ischemic heart disease (39.5%) were conducted most frequently, followed by studies of hypertension (22.4%), cerebrovascular disorders (18.4%), and heart failure (11.2%). No clear trend was observed between target disease and trial design.

**Table 2:** Numbers of trials by target disease and trial design (RCT or non-RCT)

Target disease	Number of trials			
	
	Total		RCT*	Non-RCT*
Ischemic heart disease	60	(39.5%)	39	21
Hypertension	34	(22.4%)	21	13
Cerebrovascular disorder	28	(18.4%)	23	5
Heart failure	17	(11.2%)	12	5
Hyperlipidemia	13	(8.6%)	9	4
Diabetes mellitus	10	(6.6%)	9	1
Chronic kidney disease	7	(4.6%)	5	2
Arrhythmia	5	(3.3%)	5	0

Total	152	(100%)	108	46

* RCT: randomized controlled trial, Non-RCT: non-randomized controlled trial

### 5) Number of trials by funding sources

**Figure 2:**
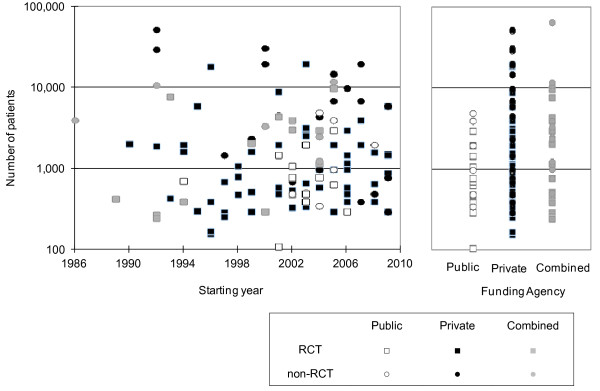
**Dot plot of starting year, funding agency, and number of participants**.

We analyzed the relationships among starting year of the trials, their numbers of participants and the types of funding agencies (Figure 2). 'Public' funding agencies include governmental organizations such as the Ministry of Health, Labour and Welfare, and 'Private' agencies include non-governmental or civilian organizations such as pharmaceutical companies.

Most of the larger-scale studies were funded by private organizations. The number of public funded studies increased from the latter half of the 1990s. Trials with combined funding sources were conducted until 2005, but no combined funding trials were conducted after 2006. We consistently found that the majority of large-scale clinical trials were privately funded. In particular, most large studies (10,000 or more participants) were privately funded.

Summary statistics of the numbers of participants by funding source are shown in Table 3. The median number of participants in publicly funded trials was 762, that in privately funded trials was 1,000, and that in trials with combined funding sources or other sources was 2,100.

**Table 3:** Summary statistics about numbers of participants by funding source

Parameter	Number of trials			
	
	Public	Private	Combined	Total
Number of trials (N) *	20	95	29	144
Mean	1,257	3,948	5,062	3,799
Standard deviation	1,340	7,959	12,064	8,463
Median	762	1,000	2,100	1,007
Max	5,000	53,000	65,434	65,434

* Eight multi-national trials where the numbers of Japanese participants was not known were excluded.

### 6) Number of trials by presence or absence of publication

We searched for publications arising from each trial in medical journals (including abstracts of medical or scientific congresses). Overall, 60.5% (92/152) of the trials were published (Table 4). Of the published trials, 70 of 92 were RCTs and 22 were non-RCTs. 39.5% (60/152) of the trials were not published. Of these 60 trials, 39 trials were RCTs and 21 were non-RCTs. However, trial designs and/or results in 33 out of 39 RCTs and 14 out of 21 non-RCTs were found in clinical trial registries, such as clinicaltrials.gov and the UMIN Clinical Trials Registry. Six RCTs and seven non-RCTs were not disclosed anywhere.

**Table 4:** Number of trials by presence or absence of publication in medical journals

Publication status	Number of trials (N = 152)		
	
	RCT*	Non-RCT*	Total
Published trials	70	22	92 (60.5%)

Unpublished trials	39	21	60 (39.5%)
Completed but unpublished trials	14	8	22
Ongoing unpublished trials	25	13	38

* RCT: randomized controlled trial, Non-RCT: non-randomized controlled trial

## Discussion

We found a total of 152 trials that met our criteria for 'large-scale clinical trials' examining cardiovascular diseases in Japan. Sixty-four trials were found on 30 November, 2004, 53 trials were additionally found on 25 February, 2007, and 35 additional trials were found on 25 July, 2009.

72.4% (110/152) of the trials were RCTs, and 27.6% (42/152) were non-RCTs. Only 14 (9.2%) trials were large-scale cardiovascular clinical trials involving 10,000 or more participants, while there were 65 trials (42.8%) with less than 1,000 participants. Thus, larger clinical trials in Japan were found to be relatively rare. We propose two possible causes for this finding: first, it may be perceived that conducting such large clinical trials is not necessary for events that are considered to have a high incidence of mortality and morbidity. Second, limited funding and human resources may limit the feasibility of large-scale clinical trials.

In trials with larger sample sizes, the proportion of non-RCTs was higher than among trials with smaller sample sizes. This finding is in accord with our proposal that large-scale clinical trials may be restricted because of limited funding and human resources.

We compared trial designs and the number of participants by search date (Table 5). This analysis revealed that the proportion of trials of cardiovascular diseases in Japan with smaller sample sizes increased over time. Furthermore, the ratio of RCTs to non-RCTs was greater among the small-scale trials than among the large-scale trials.

**Table 5:** Numbers of trials by number of participants and trial design (RCT or Non-RCT) by registration date

Number of participants	Number of trials					
	
	Until 30 November, 2004 (N = 63)		From 1 December to 25 February, 2007 (N = 53)		From 26 February to 25 July, 2009 (N = 152)	
	
	Total n (%)	Number of RCT* n (% vs Total)	Total n (%)	Number of RCT* n (% vs Total)	Total n (%)	Number of RCT* n (% vs Total)
≥10,000	6 (9.5%)	1 (16.7%)	8 (15.1%)	3 (37.5%)	0	NA

1,000-10,000	29 (46.0%)	23 (79.3%)	26 (49.1%)	14 (53.8%)	9 (33.3%)	9 (100%)

<1,000	28 (44.4%)	24 (85.7%)	19 (35.8%)	19 (100%)	18 (66.7%)	10 (55.6%)

* RCT: randomized controlled trial

After 2001, the number of large-scale clinical trials markedly increased to more than 10. In 2004, the number of large-scale clinical trials peaked, with 16 trials started within a year. In 2007, the number suddenly decreased to seven per year, then increased again after 2007. This pattern may be related to changes in the climate of public opinion regarding financial disclosures revealing relationships between study sponsors and funding agencies. Some reports in the mass media in Japan regarding private industry funding to academic sponsors without advance disclosures in 2007 and 2008 might have affected this tendency.

Ischemic heart disease (39.5%) was the most common target disease for large-scale clinical trials in Japan, followed by hypertension (22.4%), cerebrovascular disorders (18.4%), and heart failure (11.2%). Large-scale clinical trials were conducted for not only lifestyle-related chronic diseases but also for acute diseases such as myocardial infarction.

Our evaluation of changes in target diseases is shown in Table 6. The number of trials targeting ischemic heart disease gradually decreased over the study period, although it remained the major target disease in 2009. The numbers of trials targeting hypertension and cerebrovascular disorders decreased over the study period. On the other hand, the number of trials focusing on chronic kidney disease markedly increased after 2007. This was not a target disease in any trials before 2007, but seven trials on chronic kidney disease were started after 2007. This may reflect a heightened awareness of chronic kidney disease as a major risk factor for cardiovascular events [15].

**Table 6:** Numbers of trials by target disease and registration date

Target disease	Number of trials		
	
	Until 30 November, 2004 (N = 64)	From 1 December to 25 February, 2007 (N = 53)	From 26 February to 25 July, 2009 (N = 35)
Ischemic heart disease	30 (46.9%)	20 (37.7%)	10 (28.6%)
Hypertension	19 (29.7%)	13 (24.5%)	2 (5.7%)
Cerebrovascular disorder	12 (18.8%)	14 (26.4%)	2 (5.7%)
Heart failure	8 (12.5%)	5 (9.4%)	4 (11.4%)
Hyperlipidemia	6 (9.4%)	2 (3.8%)	5 (14.3%)
Diabetes mellitus	4 (6.3%)	1 (1.9%)	5 (14.3%)
Chronic kidney disease	0	0	7 (20.0%)
Arrhythmia	3 (4.7%)	1 (1.9%)	1 (2.9%)

We analyzed the relationship among the starting year of trials, the number of participants and the types of funding agencies. 'Public' funding agencies included governmental organizations such as the Ministry of Health, Labour and Welfare, and 'Private' funding agencies included non-governmental or civilian organizations such as pharmaceutical companies.

Most of the larger-scale trials were funded by private organizations, such as pharmaceutical companies. Several publicly funded trials were started in 1998, but few publicly funded trials were conducted after 2005. Trials with combined funding sources were conducted until 2005, but no such trials were started after 2006. The majority of large-scale clinical trials were consistently privately-funded. In particular, most studies with 10,000 or more participants were privately funded.

The median number of participants in public funded trials was 762, that in private funded trials was 1,000, and that in trials with combined funding sources or other sources was 2,100. This result suggests that it is possible to conduct larger scale clinical trials with private funding, but that it may be difficult to conduct studies of this size with funding from public sources. This trend was similar to the global trends for 2000-2005, where the median sample sizes of clinical trials funded by not-for-profit organizations and by for-profit organizations were 421 and 1486, respectively [4].

Thirteen trials (six RCTs and seven non-RCTs) did not disclose their designs or results at all. When we examined the changes in numbers of 'unpublished trials' in Table 7, the number did not decline between 2004 and 2009, resembling the global situation in which 54% of the trials were unpublished [16]. Among 12 completed trials with more than 10,000 participants, of which five were industry funded, 10 trials (83.3%) had been published. On the other hand, 37 (72.5%) of 51 completed trials with less than 1,000 participants had been published. When we counted the numbers of 'unpublished' trials by the type of funder, seven trials were self-funded and six were industry-funded. This means we may require different approaches to improve the current problems. For example, improving the awareness of the importance of publishing results would be effective for the sponsors of self-funded trials, while issuing guidelines to force disclosure would be appropriate for the industry-funded trials. Japan established a clinical trial registry system in 2005 [17]. Three systems, i.e. UMIN Clinical Trials Registry; Japan Pharmaceutical Information Center (JAPIC); and Japan Medical Association, Center for Clinical Trials (JMACCT) were incorporated into the Japan Primary Registries Network in 2008. As mentioned by the ICMJE in 2004 [18], the Declaration of Helsinki revised in 2008 [19] and the CONSORT declaration in 2010 [20], the disclosure of trial protocol summaries and results is important to avoid publication bias. Therefore, the situation in Japan is currently problematic.

**Table 7:** Number of trials by presence or absence of publication in medical journals by search date

	Number of trials		
	
	As of 30 November, 2004 (N = 64)	As of 25 February, 2007 (N = 117)	As of 25 July, 2009 (N = 152)
Published trials	45 (70.3%)	67 (57.3%)	92 (60.5%)

Unpublished trials	19 (29.7%)	50 (42.7%)	60 (39.5%)
Completed but unpublished trials	9	17	22
Ongoing unpublished trials	10	33	38

## Conclusions

To minimize the bias caused by funding sources, entirely publicly funded trials should be conducted by 'neutral' investigators. However, this is difficult because of limitations in the financial resources necessary for conducting large-scale clinical trials in Japan. This appears to be why most of the trials revealed by our search were funded by private industry.

Some sponsors required more than one funding source. This finding indicates that some sponsors were concerned with obtaining sufficient funding for large-scale clinical trials. However, in some large-scale trials, the relationships between sponsor and funding agencies were not clear. Some sponsors did not disclose information about trials, although this publication policy may have changed. We propose that all sponsors of clinical trials should register trials and disclose their funding sources before the enrolment of participants, and publish their results after the completion of each study to improve the quality of clinical trials. For this purpose, improving the sponsors' awareness of the importance of publications and issuing guidelines to mandate the disclosure of funding sources can offer the solutions to these problems.

## Competing interests

HS is an employee of Novartis Pharma K.K. The Department of Drug Policy and Management, Graduate School of Pharmaceutical Sciences, The University of Tokyo, is a department endowed by Towa Pharmaceutical Co., Ltd., one of the leading manufacturers of generic drugs in Japan.

## Authors' contributions

HS and KU searched the clinical trials for the study, participated in analyzing the research and drafted the manuscript. KT searched the clinical trials for the study and helped to draft the manuscript. All authors read and approved the final manuscript.
